# The Impact of Leprosy on Marital Relationships and Sexual Health among Married Women in Eastern Nepal

**DOI:** 10.1155/2016/4230235

**Published:** 2016-03-07

**Authors:** Anna T. van 't Noordende, Wim H. van Brakel, Nandlal Banstola, Krishna P. Dhakal

**Affiliations:** ^1^Netherlands Leprosy Relief, Amsterdam, Netherlands; ^2^Disability Studies in Nederland, VUmc, Amsterdam, Netherlands; ^3^Netherlands Leprosy Relief, P.O. Box 25270, Kathmandu, Nepal

## Abstract

*Background*. Leprosy is one of the most stigmatized diseases known today. The stigma surrounding leprosy can be a major burden and affects many dimensions of a person's life, including intimate relationships. We aimed to investigate the experiences of women affected by leprosy regarding marital life and sexuality, comparing these to the experiences of women with other physical disabilities and to those of able-bodied women in South-East Nepal.* Methods*. This study used a qualitative approach and a cross-sectional, nonrandom survey design. Thirty women underwent in-depth interviews about their marital and sexual relationship by means of a semi-structured interview guide. These thirty women included ten women affected by leprosy, ten women with other physical disabilities, and ten able-bodied women living in South-East Nepal.* Results*. We found that many women faced violence and abuse in their marriages. However, women affected by leprosy appeared to face more problems with regard to their marital and sexual relationships than women with physical disabilities and able-bodied women. Some of these related to the fear of leprosy.* Conclusions*. Further research is recommended to investigate the extent of this problem and ways to ameliorate the situation of the affected women. Education and counselling at diagnosis may help prevent many of the problems reported.

## 1. Introduction

Leprosy is one of the most stigmatized diseases known today [1–4]. Leprosy patients may face the effects of stigma and different forms of discrimination, such as rejection, abuse, divorce, or loss of employment, leading to reduced self-esteem and loss of respect from their communities [4–6]. Interpersonal relationships, social status, mobility, and dignity suffer [5, 6] and may cause anxiety, depression, emotional stress, isolation, and suicide or attempted suicide. Multiple studies found women to be more affected by leprosy and its stigma than men [3, 4, 6–9]. Stigma may aggravate existing inequalities due to age, gender, and social class [10]. Often, the social and psychological complications due to leprosy remain even after the medical treatment is finished. The psychosocial consequences a person has to bear after being diagnosed by leprosy are often heavier than the physical consequences that may occur [11, 12].

An important example of a social complication of leprosy is the effect of leprosy on marital relationships. A qualitative study on the psychological needs of men and women with leprosy in South Africa found that one-third of leprosy patients had been abandoned by their spouses [13]. Try found that stigma of leprosy has an effect on marriage. She states that “it is clear that in Maithili and Nepali culture, it is undesirable to marry someone who has been or is affected by leprosy” and “the prevalence of visual signs of leprosy is important [this time in] affecting the opinion of prospective partners in arranged marriages” [7]. Adhikari and colleagues, who did a cross-sectional study in Nepal among community members unaffected by leprosy, found that 48% of the community members thought that people affected by leprosy would encounter marital problems [12]. However, not much is known about the nature of the effects of leprosy on ongoing marriages and even less is known about the effects on sexual relationships and perceptions of sexuality and reproductive health.

In Nepal, as in most cultures in the Global South, marriage is considered very important [14]. Definitions of marriage may vary among Nepal's 60 ethnic groups [15]. According to Lamichhane and colleagues, however, overall “women are expected to play a subordinate, submissive, and more conservative gender role in marital relationships especially in rural areas” [16]. In particular in rural areas, early marriage is quite common [15, 17–20]. Even though there is a slow shift from arranged marriages to love marriages, arranged marriages are still predominant in Nepal and individual choices are subordinate to relationships and agreements between families [16–18, 21]. In case of arranged marriages, especially when they happen at a young age, premarital romantic relationships often do not happen. For the majority of Nepali women, the onset of sexual activity occurs within marriage [18]. Closely linked to the common practice of arranged marriages, marriage in Nepal occurs at a rather young age, with a median age at first marriage of 16.5 years for women born in the late 1970s [17].

Sexuality is closely linked to marriage and has long been avoided as a study topic because of the taboo associated with discussing sexuality. For this reason, the topic was difficult to address [22]. To our knowledge, no research has been done on the effects of leprosy on sexual relationships and perceptions of sexual health. However, a number of studies have investigated the effects of disability on sexual relationships. For people with a disability, sexuality is often not recognized as a legitimate form of pleasure and an expression of love [22]. McCabe and Taleporos, who studied predominantly people with a spinal cord injury, cerebral palsy, and acquired brain injury, found that having a physical disability leads to increased negative feelings, including a belief of being less sexually attractive than people without a disability and the feeling that people with a disability are limited in expressing their sexuality [23]. The latter are generally less satisfied with their romantic relationships than their able-bodied peers [24, 25]. People with a disability are sometimes viewed as asexual [22, 26, 27]. It is often inaccurately thought that people with disabilities lack the desire, ability, and capacity to be sexually active [27, 28]. According to Nosek and colleagues, having a mobility-related disability limits the opportunity for sexual activity. They state that “women with disabilities reported significantly lower levels of sexual activity, sexual response, and satisfaction with their sex lives” [25]. A lack of privacy, dependence on others for care, and inaccessibility to homes and meeting places also make it more difficult for people with a disability to maintain sexual relationships [29].

Infectious diseases still constitute a significant proportion of the total disease burden in Nepal. Leprosy is one of the neglected tropical diseases endemic in the country. In 2014, 3,046 new cases were registered [30]. The distribution of leprosy is not equal in Nepal. The Terai districts account for over 83% of cases [31]. In addition to those currently on treatment, many thousands of people live with residual leprosy-related disabilities, many of which are aggravated by social stigma which is still very strong.

The scanty evidence that is available indicates that leprosy may severely affect relationships, to the extent that even divorce is not uncommon in marriages in which one spouse develops leprosy [5, 13]. Given the fear of contagion that surrounds leprosy, it is likely that divorce is only the tip of the iceberg and that many problems in marital and sexual relationships go unnoticed. This study aimed to investigate the experiences of women affected by leprosy regarding marital life and sexuality, comparing these to the experiences of women with other physical disabilities and to those of able-bodied women in South-East Nepal. This study focused on women, because women are often more severely affected by leprosy and its stigma than men [3, 4, 6–9].

## 2. Methods

### 2.1. Study Design

This study used a cross-sectional, nonrandom survey design with a qualitative approach.

### 2.2. Study Site

This study was conducted in the Eastern Terai region of Nepal.

### 2.3. Study Population

Three groups of people were included in the study:

1. Women affected by leprosy, with and without visible impairments.
2. Non-leprosy-affected women with visible impairments.
3. Able-bodied, healthy women.

The first group consisted of women who had completed their leprosy treatment and women who still received treatment. Of the women who had a disability resulting from leprosy, the disabilities were graded using the grading system of the WHO, which grades impairments in eyes, hands, and feet [32]. Each hand, foot, and eye (left and right) is assessed and graded on its own. Either the maximum grade or the sum of the six grades is used as indicator of the severity of impairment. In this study, the women affected by leprosy had to have at least a grade 1 impairment. Both women with grade 1 and women with grade 2 impairments were included, to explore both problems resulting from physical appearance and problems resulting from the diagnosis of leprosy itself.

The second group consisted of women with a visible physical impairment. Included were women with an impairment obvious to the community based on appearance and/or those with limited functioning. Examples of visible physical impairments include people with neurological impairments requiring mobility aids and people with severe burn scars. Both women with congenital impairments and women who acquired an impairment later in life were included.

The third group served as “control” group. Able-bodied, healthy women were interviewed, to try and distinguish issues resulting from cultural practices, and lack of knowledge or awareness, from those caused by disability or leprosy.

### 2.4. Study Sample

In total, 10 women affected by leprosy, 10 women with a visible physical impairment, and 10 able-bodied women were included. Of the women affected by leprosy, 6 had a grade 1 impairment and 4 had a grade 2 impairment. All participants lived in the Eastern Terai region of Nepal, in Jhapa, Morang, Saptari, or Sunsari districts. If women indicated a need for support or counselling, they were referred to the Biratnagar Leprosy Referral Centre.

### 2.5. Sampling Methods

Because we wanted to interview women with specific characteristics, participants were selected using purposive sampling. All women included in the study had to be married and had to be between the ages of 18 and 50. Excluded were women with a mental illness that interfered with their ability to undergo an in-depth interview, widowed women, and women whose husbands did not know they have or have had leprosy. The participants were contacted through the Netherlands Leprosy Relief (NLR) network in the Eastern Development Region of Nepal and through local health posts. Local health posts in the study area were visited to check whether there were leprosy-affected women that met the inclusion criteria. The records of the NLR-supported referral clinic in Biratnagar were also checked. Both the women with disabilities and the able-bodied women were selected based on their similarity to the leprosy-affected women, mainly in age and living area. Women with disabilities were identified through local Disabled People's Organisations (DPOs).

### 2.6. Data Collection

Cross-sectional data were obtained from in-depth interviews. Participants underwent in-depth interviews about their marital and sexual relationship by means of semistructured interviews. Data were collected between March and June 2014. The interview guide used consisted of four themes: sense of self, marital relationship, knowledge and awareness of sexual and reproductive health, and sexual relationship. The interview guide was developed based on a literature review and on discussions with leprosy specialists. It was translated in Nepali. The translation was thoroughly checked by translating the instrument back into English. Some questions were translated multiple times using different interpreters, to ensure that the meaning of the original English version was retained.

Taking into account the sensitivity of the topic, participants were interviewed by a local, married female interpreter in their home, or at a private, safe space near their home. The interpreter had several years of experience working for NLR in the area of research. The interpreter had experience with and knowledge of working with people affected by leprosy and qualitative data collection. To minimise interobserver variation, all interviews were conducted by the same interviewer. The interview guide and the interview technique were tested by means of pilot interviews. Prior to the pilot interviews, the interpreter received an interview training of several days in which she did role plays and was provided with feedback by the researcher. The researcher herself was not present during the interviews to prevent discomfort on the side of the interviewee. Interviews of 30–70 minutes were conducted in the local language and were audio recorded.

### 2.7. Data Analysis

The recordings were translated, transcribed, and analysed using open coding and content analysis. The interviews were transcribed in English by the interpreter and discussed with the researcher to help put issues in perspective and context. The software programme “MAXQDA” was used to assist in analysing the data. All information in the transcripts was coded by the lead author (AvtN). Open, inductive coding was done in MAXQDA, where similar phrases with recurring themes were coded. All codes with supporting quotes were then clustered together in different tables, ordered by subquestion to get an overview of responses and to identify connection between codes and themes.

### 2.8. Ethical Considerations

Prior to the in-depth interviews, participants were fully informed about the nature and objective of the study and of confidentiality of the data. Written consent for participation in the study was obtained from each participant. Ethical approval was sought and obtained from the Nepal Health and Research Council.

## 3. Results

### 3.1. Characteristics of the Study Sample

Thirty women were included. The mean age was 35 years for the women affected by leprosy (range: 22–50 years), 36 for the women with physical impairments (range: 24–50 years), and 36 for the control group (range: 24–50 years). Assessment of impairments using the WHO's grading system for disabilities resulting from leprosy classified six women as grade 1 and four as grade 2. Three women still received treatment for leprosy, whereas seven women were released from treatment.

All the women (*n* = 30) who participated in this study were still with their husbands. Ethnicity was categorized into four groups: (i) Brahmin/Chettri, (ii) Dalit, (iii) Tribal, and (iv) Other. Brahmin/Chettri accounted for the biggest group (*n* = 16). The majority of women were Hindu (*n* = 26) and lived in rural areas (*n* = 21). Most marriages were arranged (*n* = 20). Love marriages occurred mostly among the women with physical disabilities, with five women having a love marriage. Except for one participant who had upper back problems, all participants with a visible physical disability had impairments related to their feet and legs, observable in walking. Except for three women, all had at least one child. Three women, one in each group, were visibly pregnant at the time of the interviews. More than half of the women (*n* = 17) indicated they did not work outside the home. No demographic data was collected about the husbands of the women included in the study.

### 3.2. Additional Information Concerning Leprosy-Affected Women

For five women affected by leprosy, all contacts, husband, neighbours, and relatives, knew they had leprosy, for two, only the husband knew, and for three, only a few people who were very close knew they were affected by leprosy. It seemed that the cause of their disease was not always well known. Four out of ten women did not seem to know the real cause of their disease. They also had misconceptions about the transmission of the disease. Once their treatment was finished, these misconceptions no longer applied. One woman told us,

*…Before there were problems, I did not give them [family] food which I had taken. I was worried that it would transfer to them….* (Woman affected by leprosy, age 33)

Another woman said,

*…My husband is afraid that it transmits through respiration, so he does not want to tongue kiss for seven months….* (Woman affected by leprosy, age 22)

In the quotes presented, women mostly referred to their situation* after* contracting leprosy.

### 3.3. Results Regarding Marriage, Sexual Relationships, and Sex Education Applicable to All Women

Most of the thirty women who were interviewed (*n* = 26/30) indicated that being married is important in their community and to themselves also. Sexual relationship, an important part of marriage, seemed to be of mixed importance. Six women from the control group indicated that sex is an important part of their relationship. For the women with a disability, five women considered it important, five did not. Six of the women affected by leprosy said they did not find sex important. Except for two women, all women indicated that sex is important to their husbands. Some women (*n* = 4/30) thought that their opinion on whether sex is important is not really of importance. According to them, what* they* think is important does not always matter. They are supposed to be ready whenever their husbands are ready, as the following quotes illustrate:

*…Yes, it is also important for me, but our importance has no value. We cannot express our feelings even with our husband….* (Woman affected by leprosy, age 26)

*…It is not necessary how important it is for us because whenever our husband is ready we should be ready….* (Woman affected by leprosy, age 50)

*…It is not so important for me because of my condition, but I used to be ready for my husband and I do not mind, after all he is my husband….* (Woman with physical disability, age 48)

Most women (*n* = 24/30) did not receive sexual education in school or through a health post, or at least not before marriage. Only six women said they received sexual education in school, mostly mentioning grade eight, nine, or ten. All of the women who mentioned they had had sexual education had received higher or secondary education. Other women mentioned they received some form of sexual education through the health post or from a neighbour or relative. Many women (*n* = 15) mentioned receiving sexual education through TV or radio.

### 3.4. Factors Affecting the Marital Relationship of Women

#### 3.4.1. Positive Factors

Several factors may influence the marital relationship of the women interviewed. Factors reported to have a positive effect on their marital relationship were love, harmony, and understanding each other, money or property, and sex. Two women said:

*…I think sex is the ultimate factor for a couple to stay happy….* (Woman with physical disability, age 43)

*…The most important is trust, love and understanding….* (Woman affected by leprosy, age 32)

#### 3.4.2. Negative Factors

Factors reported to have a negative influence on marriage are the (alcohol) drinking habit of the husband, a negative attitude of family members or others towards the woman, misunderstanding between husband and wife, and an unsupportive husband. Furthermore, two women with leprosy indicated that they felt that people were more distant since they knew they are affected by leprosy. Some examples are given below:

*…I used to stay with my father and mother in law in their house. My sister in law was very rude, she used to come to the house as she was married and tell unnecessary things to my husband. My husband used to listen to her and be angry with me….* (Woman from control group, age 32)

*…When I was diagnosed with leprosy I felt that my husband's behaviour had changed, he did not share anything with me and he pretended to be busy with work. But actually he was trying to be far away from me…. * (Woman affected by leprosy, age 33)

*…Before there were problems, when my father and mother-in-law knew about my disease, they hesitated to talk to me and come near me….* (Woman affected by leprosy, age 33)

However, the alcohol problem of the husband, if present, seemed to be the biggest problem. This was mentioned by all three groups, but was most frequently experienced by the women affected by leprosy. Twelve out of the thirty women had a husband with an alcohol problem. Of these women, three women were in the control group, four women had a disability, and five women were affected by leprosy.

When asked about the possibility of the husband of someone with either a disability or leprosy marrying and taking on a second wife, six women said they had never heard of this, 17 women said they had heard of this but had not experienced this themselves, four women indicated that only certain types of people do this, and two women affected by leprosy said they had experienced this themselves. The husband of one of these women had taken a second wife, because of her leprosy. Another husband sent his wife away to her parents' house and then had several affairs. Some women with a disability pointed out the difference between men and women when it comes to remaining faithful to their marriage. One woman said, about taking on a second wife,

*…If the husband has a disability then they marry a common woman or if he becomes disabled after marriage, then his wife stays with him, but if the woman is disabled then she cannot marry a common man and if she becomes disabled then the husband brings another wife....* (Woman with physical disability, age 32)

*…I feel that, if the problem that my husband has had had happened to me, my husband would have brought a second wife. But I am a woman so I cannot do so and I love him. Sometimes I used to be angry with myself for my condition….* (Woman with physical disability, age 32)

### 3.5. Factors Affecting the Sexual Relationship of Married Women in Nepal

#### 3.5.1. Positive Factors

There are positive and negative factors that influence the sexual relationship of women. Factors that may have a positive influence include loving each other and being emotionally engaged, understanding each other, obeying the husband and/or giving priority to him, and the husband not drinking alcohol. Two women said,

*…If sexual intercourse is a mutual understanding then we can get pleasure….* (Woman from control group, age 32)

*…Love helps for the good sexual relationship with my husband….* (Woman affected by leprosy, age 35)

#### 3.5.2. Negative Factors

Factors that may have a negative influence on the sexual relationship of married women include the alcohol problem of the husband, being forced by the husband to have sex, disagreeing with the husband, and reduced interest in having sex on the side of the wife. Only 11 women indicated not having any sexual problems. The husband drinking alcohol and, with that, sexual abuse were most often mentioned. One woman said about this,

*…He has the bad habit of drinking alcohol. He wants every time when he is drunk. I feel so irritated but what can I do, we think of our husband as God and we should obey him….* (Woman affected by leprosy, age 26)

About being sexually abused, two women said,

*…When I do not want to have sexual intercourse, my husband forces me. He scolds me “I used to earn money, bring food for you all but you do not want [to have sex], then get out of the house!” Sometimes he raised a hand on me. So I have to be near and close and have sex with him….* (Woman affected by leprosy, age 32)

*…He never asks about my health and forces me to have intercourse. I feel like I am a doll to him….* (Woman with physical disability, age 33)

Of the women facing sexual abuse, three women were in the control group, three women had a disability, and five were affected by leprosy. The only two Muslim women included in this study both faced sexual abuse. Furthermore, three of the four women who had a leprosy-related grade 2 impairment were being sexually abused by their husbands. In addition, of the women who had a husband with an alcohol problem (*n* = 12), all but one faced sexual abuse by their husbands, as illustrated below:

*…There were problems, he used to drink alcohol and come near me and force me to have sexual intercourse….* (Woman affected by leprosy, age 41)

*…When he drinks alcohol and comes I feel irritated and he forces me to have sexual intercourse with him….* (Woman from control group, age 32)

Sexual abuse and alcohol abuse seemed to go hand-in-hand with violence. Many (*n* = 7/10) women were either beaten or threatened to be beaten if they do not obey. This also became clear when talking about what happens if they do not agree with their husbands:

*…If I refuse him he scolds me and raises a hand on me….* (Woman with physical disability, age 33)

*…If the husband likes to have sex, then we have to give, if not he certainly beats me….* (Woman from control group, age 50)

*…I have to give him everything he wants even when I am not feeling well, because he gets angry if I refuse to give. He warns me that he'll have sexual pleasure with another girl if I cannot give pleasure….* (Woman from control group, age 28)

Five women affected by leprosy, of whom three also faced sexual abuse, faced additional problems while receiving treatment or when they were first diagnosed. These problems disappeared later on and were not experienced at the time of the interviews. They included having no intercourse at all due to fear of transmission of the disease, experiencing more distance and sometimes sleeping in separate beds while taking medicine:

*…At first when he knew that I was affected by leprosy he did not sleep with me. He used to scold me for no reason. Once he came to the Biratnagar clinic with me, he asked the doctor about the sexual relationship. He was told that it does not transfer to him so he started having sexual intercourse with me again….* (Woman affected by leprosy, age 32)

One woman did not want to talk about the problems she had before,

*…Before I had very bad problems, but now there is no effect, I do not want to remember the past and talk about that….* (Woman affected by leprosy, age 35)

## 4. Discussion

We found that many women experience marital problems and/or sexual abuse, regardless of their leprosy or disability status. In addition, we found clear indications that leprosy may influence the marital and sexual relationship of married women in various ways. This included significant problems during treatment, which is often a full year, such as having no intercourse at all due to fear of contagion, experiencing more distance from close others, and husband and wife sleeping in separate beds. Others were being abandoned or sexually abused by the husband even after treatment. These problems may be due to the negative attitudes surrounding the diagnosis of leprosy. Other studies found stigma to have negative consequences for persons affected by leprosy, leading to discrimination [4–6, 33], problems in interpersonal relationships, and problems with social status [5, 6].

Women affected by leprosy seem to face most problems when first diagnosed or while receiving treatment. The above problems may have been caused or aggravated by the fact that almost half of the women did not seem to know the cause of their disease and how leprosy is transmitted. This may have been true for their spouses also. Thilakavathi and colleagues, who conducted in-depth interviews with 72 leprosy-affected men and women, of whom 48 were married, found that a few participants did not sleep in the same room as their spouses, but they did not elaborate on this [34]. They also found that most of their interviewees lacked basic knowledge on the transmission and cause of leprosy.

Leprosy-affected women are sometimes abandoned by their husbands. This happened to two women with grade 2 impairments due to leprosy in our study. Qualitative evidence suggests that women are more likely to be deserted by their spouses than men, but conclusive evidence is not yet available [7]. Research in South-East Nepal found that, of the nine men and ten women interviewed, three husbands had left their leprosy-affected wife and one wife had left her leprosy-affected husband [7]. The reason for the spouse leaving was the other spouse's diagnosis of leprosy. The separation occurred a few months after diagnosis. These findings are similar to those in our study. A study in South Africa showed that “of 23 married subjects, 9 men and 7 women had been deserted by their marriage partners because of leprosy” [13]. We cannot tell whether the frequency of divorce found by Scott [13], which is much higher than in our study, is due to sampling error, bias in the samples, or actual cultural differences, since the present study was only designed to explore the impact of leprosy, not to determine the prevalence of marital problems or divorce due to leprosy. Our findings indicate that divorce is only the tip of the iceberg of marital problems that may be due to leprosy or other causes.

An important finding is the high frequency of alcohol abuse among the husbands of the participants. This occurred in all three groups and therefore points to a more structural phenomenon in society. A larger survey using random sampling will have to confirm whether the greater frequency of alcohol abuse among the husbands of leprosy-affected women compared to the other women included in this study is real, or a result of sampling error. Jhingan and colleagues looked at alcohol dependence in Dharan, in Eastern Nepal, and found the prevalence of alcohol dependence to be 25.8% [35]. They found dependence to increase with age, peaking with 41% in the age group 45–54, compared to 10.7% in the 15–24 age group. Alcohol dependence was more than twice as common in men as in women. The findings of the present study support the findings of Jhingan and colleagues [35]. The severity of alcohol abuse in the present study is not known. Several studies found the risk of sexual abuse and violence towards women to increase when husbands are drunk or are alcoholics [36–40]. These studies were also conducted in developing countries and highlight the important role of alcohol use in sexual abuse [36–40]. The present study seems to confirm the relationship between alcohol abuse and sexual abuse: most of the women who had a husband with an alcohol problem experienced sexual abuse and all husbands who sexually abused their wives reportedly had an alcohol problem. We did not find any literature on the relationship between alcohol abuse and sexual abuse when the spouse has an impairment.

Sexual abuse by the husband occurred in all groups, but the frequency was higher among women affected by leprosy. Again, this may be due to sampling error and/or bias in subject selection. A larger follow-up study using random sampling will need to clarify this. Another study assessed the occurrence of violence against young married women aged 15–24 years in rural Nepal [16]. As many as 53% reported having experienced some form of violence in their lifetime and 46% reported experiencing sexual violence. No or little interspousal communication and low autonomy of women were associated with violence against women [16]. Other studies that investigated sexual violence against young married women found a similar prevalence (49%) [18, 41]. Lamichhane and colleagues associated women's lower status in family and society with violence against women, particularly young women in rural Nepal [16]. Also Pradhananga and Shrestha [42] and Puri and colleagues [18] stress the low status of women in Nepal. Deepak and colleagues, who looked at violence and sexual violence against persons with disabilities in India, found that 14% of their 146 participants reported experiences of sexual violence during the previous 12 months [43]. The presence of visible impairments among leprosy patients and its influence on acceptance by others have been highlighted by other studies [44, 45]. Kopparty, who looked at coping strategies of 500 families who had a leprosy-affected family member with and without disfigurement, found that “the proportion of families having patients with deformities facing problems was ten times higher (57%) than those having patients with no deformities (5.7%)” [45]. It is therefore not unlikely that women with visible impairments would experience more discrimination and abuse than leprosy-affected women without visible impairments. Their position in society may be low, possibly aggravated by alcohol abuse of the husband. This in turn may lead to sexual abuse. Furthermore, Puri and colleagues found that 8 out of 15 women who refused to have intercourse with their husbands were beaten [41]. Being beaten or threatened with violence when not obeying their husbands was also reported by women in the present study.

Our study showed that a husband often has power over his wife and that wives are expected to obey their husband or otherwise may be expected to be punished. Feelings were often not shared, because women felt their feelings and desires were not valued. These findings fit with the description of prevailing attitudes towards women described by Regmi and colleagues [46]. They stated that, in Nepal, “unequal power relations and lack of autonomy characterise the situation of married young women in many settings, the autonomy of married young women is particularly constrained” and “gender norms stress male entitlement to sex, even if forced within marriage.” Certain social roles are expected, and most of the women's roles revolve around the household [7].

An important finding was that most women had not received sexual education, or at least not before marriage. The few women who had sexual education before marriage received this sometime between grades 8 and 10 in school. Regmi and colleagues reported that “there are major gaps in receiving information, services, and skills on sexual and reproductive health issues” [46]. The Government of Nepal has introduced sexual and reproductive health education in public schools for grades six to ten and in university curricula from 1998 onwards [46]. Regmi and colleagues assert that, in reality, young people do not have good access to appropriate information on sexual and reproductive health issues. This fits with our finding that several women who were in their twenties and who had secondary education or more indicated that they did not receive sexual education, despite the fact that a sexual and reproductive health education programme had already been introduced when they were in school.

The current findings show that knowledge about leprosy and the relation between leprosy and marriage and sexual health should be addressed preventively whenever someone is diagnosed with leprosy. If at all possible, the spouse and possibly the in-laws of any newly diagnosed married patient should be included in such counselling efforts. Materials addressing these issues should be developed and should be made available for distribution in primary health centres and other health facilities where persons affected by leprosy are diagnosed and treated. However, it was evident in our study that within-marriage violence and sexual abuse of women, aggravated by alcohol abuse of the husband, occurred in all groups regardless of health or disability status. Sexual and reproductive health, freedom from violence, and freedom from discrimination are fundamental human rights that were systematically violated in the lives of many of the women interviewed. Therefore, interventions to improve sexual health and safety of married women should be designed, tested, and implemented as a matter of urgency.

## 5. Limitations of Study

The first limitation is the nonrandom sampling and small study size, as mentioned above. This, together with the specific geographic location of the present study, means that the results of the study cannot be generalized to the whole study population or beyond. Furthermore, due to time constraints, the interviews only included women. If men would have been included also, a more complete insight of the impact of leprosy on marital and sexual relationships could have been given. Another limitation was the use of an interpreter. Translating the interviews from Nepali to English may have introduced some mistakes or misinterpretations, since not all words could be translated literally.

## 6. Further Research 

The current study indicates that leprosy may influence the marital and sexual relationship of women in Nepal in several ways. Additional research is needed to gain more insight in the underlying reasons. Knowing more about the factors that influence marital and sexual relationship may help patients as well as health workers deal with marital and sexual problems and may enhance their ability to anticipate and prevent problems. It will also inform policy and interventions to reduce within-marriage sexual abuse and violence. Raising awareness of health workers concerning the risk of marital problems following a diagnosis of leprosy is essential. Simple educational materials with facts about leprosy, explaining the absence of risk of transmission once someone is being treated, would help health workers discuss these very important issues with their patients, preferably together with their spouses. On a wider scale, interventions to improve sexual health and safety of married women should be implemented as a matter of urgency. Implementation research should investigate the appropriate format and manner in which this can be done. A larger study with a random sample is needed to determine the extent of the problems identified and the added risk of being leprosy-affected or having a disability.

## 7. Conclusions

1. Many women in our study experienced marital problems and/or sexual abuse, regardless of their leprosy or disability status. Fundamental human rights such as the rights to sexual and reproductive health, freedom from violence, and freedom from discrimination are systematically violated in the lives of many of the women interviewed.
2. Women affected by leprosy faced additional problems, related to fear of the disease, negative attitudes, and discrimination on account of leprosy.
3. Knowledge on the cause and transmission of leprosy was still lacking among leprosy-affected women and their community members. Appropriate preventive and educational measures should be designed and tested to address these issues.
4. Lastly, women appear to have insufficient access to sexual education, despite programmes on sexual health education in schools and elsewhere. This should be addressed by the appropriate authorities.
